# Predicting reverse-bound peptide conformations in MHC Class II with PANDORA

**DOI:** 10.3389/fimmu.2025.1525576

**Published:** 2025-03-24

**Authors:** Daniel T. Rademaker, Farzaneh M. Parizi, Marieke van Vreeswijk, Sanna Eerden, Dario F. Marzella, Li C. Xue

**Affiliations:** ^1^ Biosystems Data Analysis, University of Amsterdam, Amsterdam, Netherlands; ^2^ van‘ t Hoff Institute for Molecular Sciences, HIMS-Biocat, University of Amsterdam, Amsterdam, Netherlands; ^3^ Amsterdam Machine Learning Lab, University of Amsterdam, Amsterdam, Netherlands; ^4^ Medical BioSciences Department, Radboud University Medical Center, Nijmegen, Netherlands

**Keywords:** reverse-bound peptides, HLA II, homology modeling, peptide-MHC, MHC II

## Abstract

Recent discoveries have transformed our understanding of peptide binding in Major Histocompatibility Complex (MHC) molecules, showing that peptides, for some MHC class II alleles, can bind in a reverse orientation (C-terminus to N-terminus) and can still effectively activate CD4+ T cells. These finding challenges established concepts of immune recognition and suggests new pathways for therapeutic intervention, such as vaccine design. We present an updated version of PANDORA, which, to the best of our knowledge, is the first tool capable of modeling reversed-bound peptides. Modeling these peptides presents a unique challenge due to the limited structural data available for these orientations in existing databases. PANDORA has overcome this challenge through integrative modeling using algorithmically reversed peptides as templates. We have validated the new PANDORA feature through two targeted experiments, achieving an average backbone binding-core L-RMSD value of 0.63 Å. Notably, it maintained low RMSD values even when using templates from different alleles and peptide sequences. Our results suggest that PANDORA will be an invaluable resource for the immunology community, aiding in the development of targeted immunotherapies and vaccine design.

## 1 Introduction

The Major Histocompatibility Complex (MHC) plays a critical role in immune responses, allowing T cells to recognize cells presenting non-self peptides, such as those from pathogens or cancer cells (1). MHC is categorized into Class I and Class II. Class I molecules, expressed across all nucleated cells, predominantly present peptides derived internally to CD8+ T cells. In contrast, Class II molecules, primarily found on antigen-presenting cells like macrophages, B-cells, and dendritic cells, present externally derived peptides to activate CD4+ T cells. As both CD4+ (2, 3) and CD8+ T cells (4) are involved in recognizing and eliminating cancer cells, understanding peptide interactions with MHC and T cells is crucial for developing targeted therapies, including personalized cancer vaccines (5).

**Figure 1:**
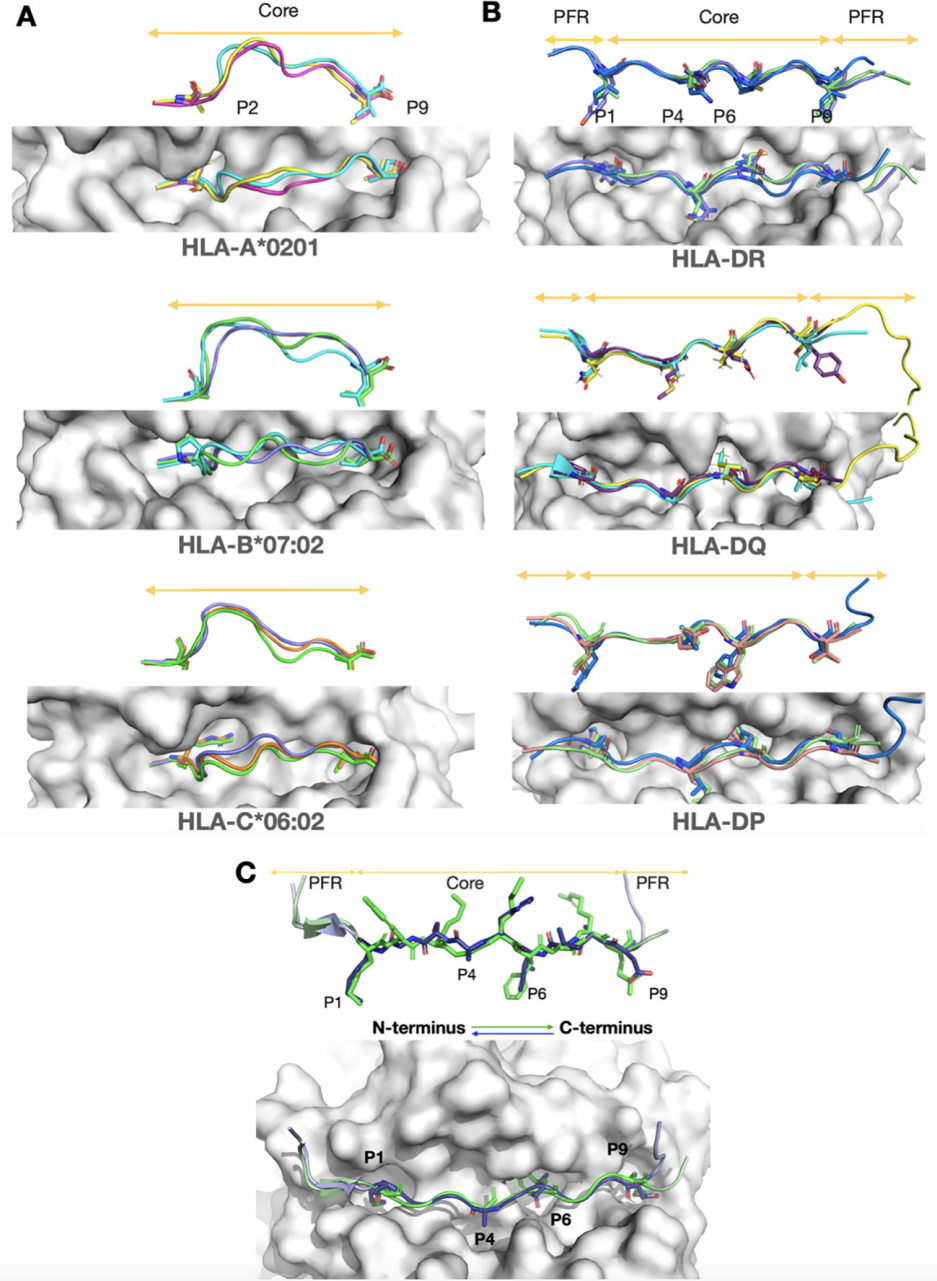
Structural insights from experimental structures of peptide flexibility in MHC Class I, and MHC Class II, and for canonical and reversed orientations. A top view and 90-degree rotated side view of the peptides are shown for each case. MHC molecules are depicted as grey surfaces, and peptides are highlighted in color. Core regions and peptide flanking regions (PFRs) are indicated by orange arrows. The main anchor residues are shown as sticks, while the rest of the peptide backbone is represented in cartoon form. **(A)** MHC class I molecules, which typically bind 9-mer peptides, are stabilized by two anchor residues positioned at both ends of the groove. Despite this stabilization, the middle region of the peptide core is highly flexible and bulges outward from the groove, as seen in the side view. This structural adaptability enables MHC class I molecules to accommodate a wide range of peptide sequences. Shown are the most common alleles from three prevalent genes: HLA-A02:01 (5H5Z, cyan; 2GUO, dark pink; 1AKJ, yellow), HLA-B07:02 (6UJ7, green; 6VMX, cyan; 7LGD, purple), and HLA-C*06:02 (8SHI, green; 5W69, purple; 5W6A, orange). **(B)** Unlike MHC class I, MHC class II peptides have a restricted core tightly anchored by four residues within the groove. Extensive hydrogen bonding between the peptide backbone and the MHC molecules further stabilizes the binding, making it largely sequence-independent (10). The PFRs are less constrained, allowing for the accommodation of longer peptides, as indicated by orange arrows. Despite variations in alpha and beta chain allele combinations, the peptide core conformation remains consistently restricted across gene types. Shown are HLA-DP (7T2A, salmon; 7T2C, white; 3LQZ, blue) for HLA-DP4 and HLA-DP2 alleles, HLA-DQ (6DIG, cyan; 7KEI, yellow; 6U3M, purple) for HLA-DQA1 and HLA-DQA1*01:02 alleles, and HLA-DR (4I5B, green; 1FYT, purple; 3C5J, blue) for DR1 and DR5 alleles. **(C)** Experimental structures of canonical (7ZAK, green) and reversed (7T6I, light purple) peptide orientations in HLA-DP are shown, with arrows indicating direction. Despite the different orientations, note that the core backbone conformation remains highly similar.

MHC Class I molecules are composed of a single α-chain paired with β2-microglobulin and feature a closed-ended peptide-binding groove that accommodates peptides usually 8–11 amino acids long. These peptides bind primarily at the termini, typically at P2 and P9, with the rest of the peptide extending outward in a flexible loop-like conformation (**Figure 1A**). In contrast, MHC Class II molecules consist of an α-chain and a β-chain, with a structurally similar yet open-ended peptide-binding groove. This open structure allows MHC Class II to bind longer peptides, generally more than 12 amino acids, with a core of about 9 amino acids tightly anchored in the groove and flanking regions extending outward (1) (**Figure 1B**). Since CD4+ T cell receptors mainly interact with the tightly bound peptide core, this part of peptide modeling is of particular interest to researchers (6, 7).

Research has demonstrated that the MHC II-bound peptide core’s backbone conformation is highly conserved, influenced by two primary factors. The first involves anchor residues at specific sites (primarily: P1, P4, P6, P9 of the core) which bind to MHC pockets (8), with binding affinity determined by the residues’ charge and size (9), and are conserved across allele-specific binding peptides (see **Figure 1B** for examples). The second factor is an allele-specific, highly conserved hydrogen bonding pattern between MHC Class II residues in the groove walls and the peptide backbone, stabilizing the backbone’s conformation (10). As concluded by Jones et al., these two factors ensure that the conformation of peptides in the MHC II complex is governed primarily by the characteristics of the MHC allele, making it independent of the (non-anchor) sequence (10).

It is no surprise, therefore, that the most accurate predictors of MHC II peptide conformations, such as the homology modeling-based PANDORA (11, 12) and AlphaFold-based methods (13, 14), are data-driven. These techniques capitalize on the conserved core peptide backbone, using a vast dataset of experimentally determined MHC II-bound peptide structures from various alleles, directly or indirectly, as templates to model new peptides. However, their strength also determines their weaknesses, as their limitations are based on the availability of known (or similar) allele complexes (15–17).

A notable example of such limitations is the case of reversed-bound peptides of MHC II, for which only two structures are currently available in the protein databank (PDB) (18). Traditionally, peptides were assumed to bind to MHC II molecules exclusively from the N-terminus to the C-terminus. Recent evidence, however, shows that some alleles can bind peptides in reverse, from the C-terminus to the N-terminus (19–22), thereby broadening the variety of peptides that can be presented. This research has particularly focused on a variety of HLA-DP allotypes found in 16% of the global human population, indicating that this is a common occurrence rather than a rare phenomenon (19). One of these studies also confirmed that virus-specific human T-cells can recognize these reversed peptides (19). Therefore, modeling these reversed-bound peptides is vital for advancing immunological research, although the current dependency on existing data presents significant challenges. This challenge was notably addressed by Mikhaylov et al., who explicitly attempted to model reversed-bound peptides using their state-of-the-art AlphaFold2-based pipeline; however, they reported their failure to do so (13).

Interestingly, both Klobuch et al. and Racle et al. noted similarities between forward- and reverse-oriented peptides within the same allele (19, 21). Both orientations use the same anchor residues and MHC II binding pockets (see **Figure 1C**), but in reversed order due to their opposite orientations. For example, if a canonical peptide contained the four anchor residues K, L, V, and E in sequence, the reversed variant would present these residues as E, V, L, and K in sequence. Additionally, structural analysis revealed that both orientations also share the conserved backbone hydrogen bonding pattern, although slightly shifted (19). These findings suggest that the information needed to model reversed peptides is already present in traditionally oriented peptides, providing a viable strategy for their accurate modeling.

In our current study, we present an update to PANDORA, which now includes a new feature for automatically generating reversed orientation of peptides to be used as templates for homology modeling. These reversed-oriented peptides, although inspired by canonical-oriented peptides, are novel, with unique structures, unique sequences, distinct phi and psi angles, and chiral centers. We validated this approach by predicting the conformations of two reversed-bound peptides of known MHC II complexes, achieving an average L-RMSD error of 0.63 Å on the peptide binding cores, which is comparable to the results from PANDORA’s modeling of canonical peptides. To the best of our knowledge, PANDORA is the first tool to model reversed-bound peptides, contributing a new capability to the field of immunological research.

## 2 Methods

We created a database of structure templates with reversed peptides by inverting the peptide backbones in the canonical MHC-II complex structures obtained from IMGT (23). These templates are used by PANDORA to assign the initial conformation to the target sequence. When these template peptides are used to model a new peptide, the target sequence is threaded onto the template structure, and its side chains are replaced and optimized through energy minimization to ensure proper geometry and interaction within the MHC binding groove. For further details on the methodology and optimization strategies employed in PANDORA, we refer readers to our previous work (11, 12).

**Figure 2:**
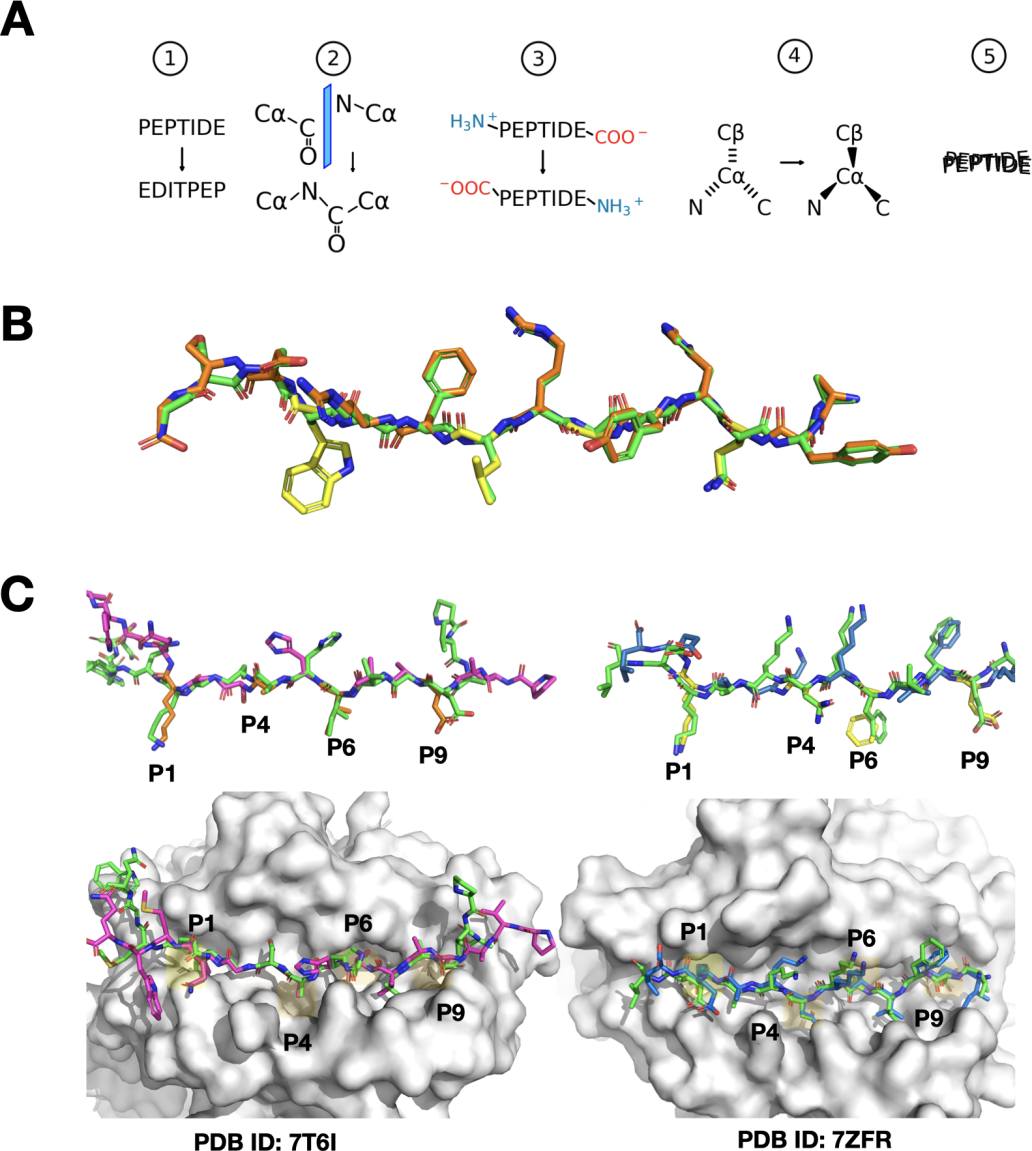
Visual overview of peptide reversal and PANDORA modeling predictions. **(A)** Schematic representation of the peptide reversal process in five steps (1): reversing the sequence numbering and order of the peptide in the PDB file without changing the locations of the residues (2), mirroring of carbonyl (C=O) and nitrogen (N) groups between the alpha carbons (CA) (3), removing and regenerating terminal groups (NH3+ and COO-) at opposite ends (4), adjusting CA chirality, and (5) performing molecular dynamics to restore geometry. **(B)** Comparison of the canonical peptide from PDB entry 1AQD (green) and its reversed variant artificially generated by our software (orange, with anchor residues separately highlighted in yellow), illustrating the initial and modified peptide structures. **(C)** Left panel: Model of the reversed peptide corresponding to the sequence from PDB entry 7T6I, using 7ZAK_reversed as the template (same allele). The modeled peptide is shown in purple, with main anchor residues highlighted in orange, while the actual X-ray structure of the peptide is depicted in green. This showcases PANDORA’s accurate modeling when using a template from the same allele. The MHC molecule is shown as a white surface, with major binding pockets shaded in yellow. Right panel: Similar to the left panel, but the model corresponds to the sequence from PDB entry 7ZFR, using 3WEX_reversed (26) as the template (different allele). The modeled peptide is shown in blue. This illustrates PANDORA’s ability to generalize to different alleles and accurately predict reversed peptide structures using templates from different alleles.

The preprocessing steps included stripping the PDBs of hydrogen atoms and replacing selenomethionine residues with methionines, which can be properly handled by the Amber14 forcefield (24). After generating the initial reversed templates, the peptide residues were renumbered in reverse order, effectively inverting the sequence. Next, we identified the individual planes formed by the carbon (C), nitrogen (N), and oxygen (O) atoms between the alpha carbons (CA) of adjacent amino acids. This step was crucial for preserving the orientation of hydrogen bonds and ensuring that these (N, C, and O) atoms remained in the original plane during the reversal process. Using each plane as a reference, we mirrored the backbone carbonyl (C=O) group with the nitrogen atom across the midpoints between the CAs (see **Figures 2A, B**). After this mirroring process, we reassigned the atoms to the correct amino acids in the PDB file, as the mirroring shifted these atoms to neighboring residues. Finally, reversing the peptide sequence required the removal of the peptide’s C-terminal (COO-) and N-terminal (N-) groups, followed by re-generating these terminal groups to the correct ends of the reversed peptide using PDBFixer (25).

The rearrangement of atoms resulted in a peptide composed of amino acids in the D-configuration, indicating a reversal of the chirality centers. To achieve the natural L-form, we mirrored the CAs across the plane formed by the N, C, and beta carbon (CB) atoms for each amino acid. Glycines were excluded from this adjustment because they are non-chiral. Similarly, we adjusted the configuration of proline residues by recalculating the CB, CD, and CG side-chain atoms using PDBFixer, resulting in the more common trans isomer.

As the translation of atoms introduced slight distortions, including slight alterations in bond lengths, we performed a short molecular dynamics run with OpenMM (25) to restore proper geometry. Before running the MD simulation, we reintroduced hydrogens using PDBFixer and then conducted a 10-step MD run with the Langevin integrator at 300 Kelvin, constraining the MHC complex while allowing only the peptide to move.

After the simulation, we renumbered the anchor residues to ensure consistency with the reversed peptide sequence. For instance, the first anchor residue in the original sequence becomes the last anchor in the reversed sequence, even though it still occupies the same binding pocket in the MHC molecule. The resulting PDB files, containing reversed peptides, were manually inspected, and stored to be used as templates for reverse peptide modeling with PANDORA. All resulting templates are given an ID with the format of PDBID_reversed, e.g., 3WEX_reversed.

## 3 Results

To evaluate the modeling and generalization capabilities of our enhanced PANDORA tool, we conducted two experiments, constrained by the availability of only two structures of reversed peptide-MHC complexes.

The first test involved modeling the reversed MHC-II complex with PDB ID: 7T6I, which includes the DPA102:01-DPB101:01 allele and a reversely-bound peptide PVADAVIHASGKQMWQ. We employed PANDORA, which automatically selected 7ZAK_reversed as the template for modeling, featuring the same alleles and a reversed peptide, DIERVFKGKYKELNK (originally KNLEKYKGKFVREID). This setup allowed us to directly compare the predicted model against the available X-ray structure. We calculated the ligand root-mean-square deviation (L-RMSD) of the binding core residues of the peptide, where L-RMSD measures the average distance between the backbone atoms of the modeled peptide and those in the X-ray structure. The core L-RMSD was 0.746 Å (see **Figure 2C**), demonstrating PANDORA’s ability to model reversed peptides with high structural accuracy, within the range of the reported core L-RMSD of PANDORA for canonical peptides binding to MHC-II (12).

In our second experiment, designed to assess the generalization capacity of PANDORA, we modeled another MHC-II complex: PDB entry 7ZFR, containing the reversed-bound peptide IEFVFKNKAKEL with the same alleles as in the first experiment (HLA-DPA102:01-HLA-DPB101:01). To challenge PANDORA with a template involving different alleles and a distinct peptide sequence, we deliberately excluded PDB entry 7ZAK from the template selection. PANDORA chose 3WEX_reversed as the template, which contains the reversed peptide FQNFAVTVK (originally KVTVAFNQF) and alleles DPA102:02-DPB105:01. The core-peptide L-RMSD for this second experiment was 0.52 Å (see **Figure 2C**), aligning with the range of reported core L-RMSD of PANDORA when modeling canonical peptide-MHC II complexes (12). This result suggests that PANDORA can effectively model reversed peptides even when using templates with different alleles and peptide sequences, achieving accuracy comparable to its performance on canonical peptides.

To provide readers with a basis for comparison, we would like to mention that, on a set of 835 peptide-MHC-I canonical-oriented complexes spanning 78 MHC types, PANDORA generated models with a median RMSD of 0.70 Å and an overall mean deviation of 0.82 Å (11). Additionally, for peptide MHC-II, PANDORA evaluated 136 experimentally determined pMHC-II structures covering 44 unique αβ chain pairs, achieving a median L-RMSD of 0.49 ± 0.27 Å (12).

## 4 Discussion and conclusion

Here we present an updated version of PANDORA, an enhanced tool for modeling MHC II-peptide interactions, now with the added capability to predict reversed peptide bindings. Our results demonstrate that PANDORA can accurately model reversed peptide interactions for specific alleles, as evidenced by the low L-RMSD values obtained in our experiments.

Although our testing was limited to two cases due to the scarcity of available reversed peptide-MHC II structures, PANDORA successfully generalized its predictions using both templates from the same and different alleles. We believe this success stems from the physical constraints shared between canonical and reversed peptides, which were explicitly leveraged in generating reversed templates. Building on the proven ability of PANDORA to generalize across various templates, as shown in previous studies (11, 12), we anticipate that this capability will extend to reversed peptides as well, ensuring robust predictions across diverse scenarios.

It’s important to acknowledge potential limitations. The most significant challenge is presented by rare alleles that lack corresponding canonical peptides in the Protein Data Bank. For these alleles, the absence of direct structural analogs can hinder the ability of PANDORA to accurately predict reversed peptide bindings.

Given the rise of many deep learning-based structure prediction models, such as AlphaFold, OpenFold, OmegaFold, RoseTTAFold, and ESMFold, to name a few, researchers should be mindful that these tools come with their own unique set of challenges when considering their use for modeling peptide-MHC complexes. One significant issue, compared to homology-based approaches like PANDORA, is that they tend to be end-to-end, leaving little control for users to adjust to their specific needs, such as the direction of the peptide, choosing which residues to use for anchoring, or excluding specific data from the modeling process. Another considerable concern is that these models are directly trained on structures in the PDB, and especially for newer models, it is likely that they have been trained on the same peptides that are used for testing, which undermines the reliability of these tests.

In conclusion, while the testing of our tool is constrained by the scarcity of reversed peptide-MHC II structures, we believe that the updated PANDORA tool will provide the immunology community with a powerful resource for modeling and analyzing reversed peptides in MHC II complexes. This capability may enhance immunotherapy and vaccine design by identifying novel epitopes, opening new avenues for the development of targeted immunotherapies and personalized vaccines. Future work will focus on expanding validation to refine predictions further.

## Data Availability

The testing data used in this study was obtained from the Protein Data Bank (PDB). Source code and data for PANDORA (version 2.1.0) are freely available at https://github.com/X-lab-3D/PANDORA. Additionally, the template database, including the reverse templates mentioned in this paper, has been uploaded to Zenodo and can be accessed at https://doi.org/10.5281/zenodo.6373630.
