# Supplementary material for: Predicting reverse-bound peptide conformations in MHC Class II with PANDORA
Source: Front Immunol. 2025 Mar 24;16:1525576. doi: 10.3389/fimmu.2025.1525576 (PMC11973093; doi:10.3389/fimmu.2025.1525576)
Supplement: Supplementary file 1 [file DataSheet1.pdf]

Supplementary: Predicting Reverse-Bound Peptide Conformations in MHC Class II with PANDORA

Daniel T. Rademaker[1,2,3], Farzaneh M. Parizi[4], Marieke van Vreeswijk[3,4], Sanna Eerden[4], Dario F. Marzella[4], and Li C. Xue[4,*]

1 Biosystems Data Analysis, University of Amsterdam, 1090 GE Amsterdam, the Netherlands

2 HIMS-Biocat, University of Amsterdam, Science Park 904, Amsterdam 1098 XH, The Netherlands

3 Amsterdam Machine Learning Lab, University of Amsterdam, Science Park 900, 1098 XH Amsterdam, The Netherlands

4 Medical BioSciences department, Radboud University Medical Center, 6525 GA Nijmegen, The Netherlands

Description of Side-Chain Recovery

In our modeling process, side-chain recovery involves threading the target sequence onto a template structure and systematically replacing side chains. These side chains are then optimized through energy minimization to achieve proper geometry and ensure optimal interactions within the MHC binding groove. The inherent flexibility of side chains enables them to adopt multiple, functionally equivalent conformations, even if the exact chi angles differ.

As shown in Figure 2 of the main paper, variations in chi angles are evident between the predicted models and the experimentally determined structures. Despite these differences, the functional integrity of the interaction remains preserved. For example, lysine side chains in both predicted and experimental structures adopt different chi angles but occupy equivalent spatial volumes within the binding pocket, correctly positioning their functional groups to form essential salt bridges.

Chi Angle and backbone torsion angles

Tables S1 to S4 provide a detailed information of backbone phi and psi torsion angles and sidechain chi angles allowing comparison between the predicted and experimental structures. Additionally, Figure S1 illustrates the distribution of chi angles for core anchor residues. Note that the peptides were rotated a bit individually to make the numbers appear most clear and do not overlap well anymore, we refer to the main manuscript Figure 2 to see superimposed peptides.

Although some deviations were observed in the sidechains, the backbone was well predicted. The backbone torsion angles (phi and psi) align closely with the experimentally determined structures, reflecting the robustness of the hydrogen bonding network and anchoring at key residues we obtained from the canonical structures. Side chains projecting outward, on the other hand, exhibited greater variability, consistent with their known flexibility.

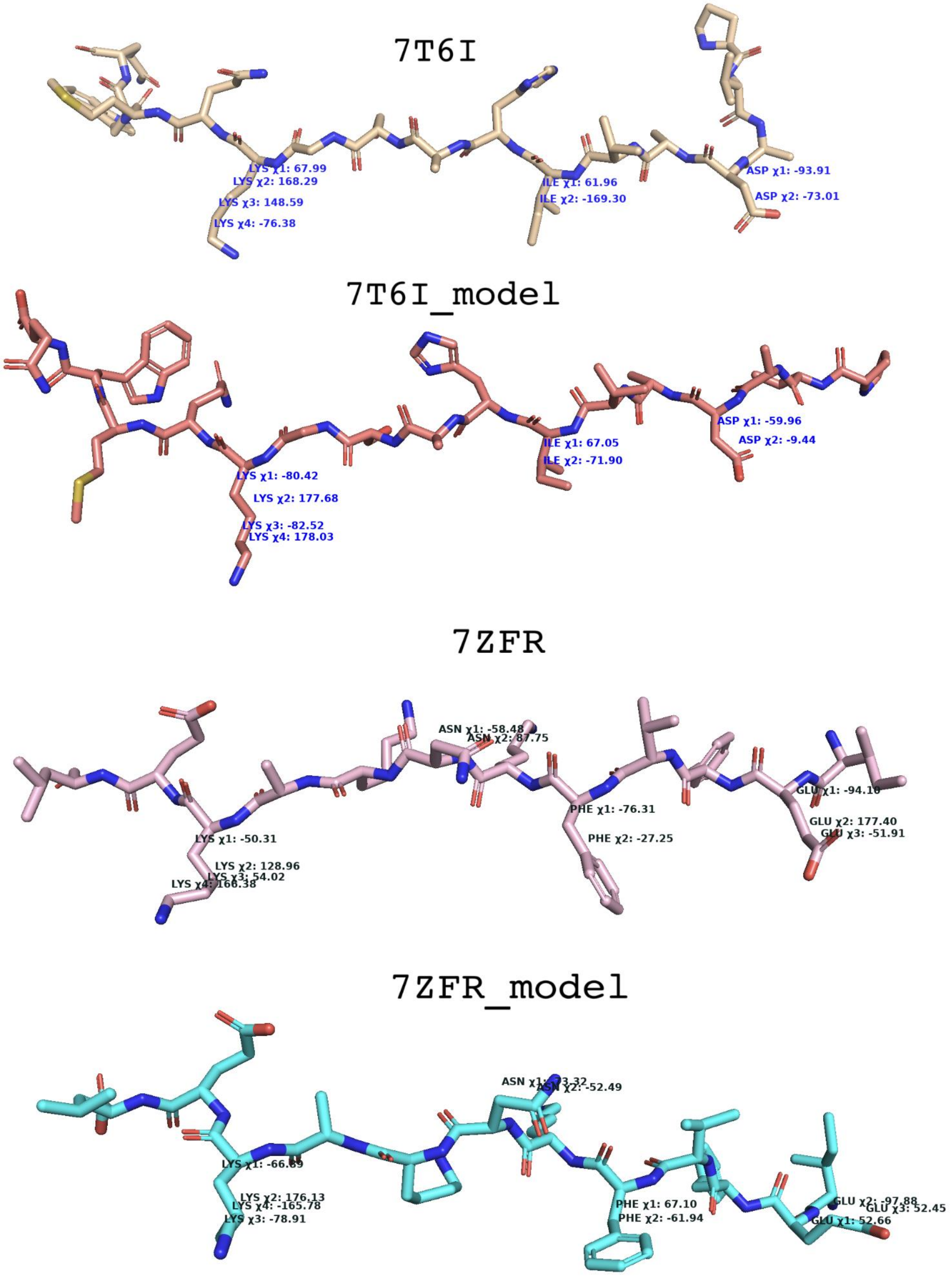

Figure S1: Visualization of Core Anchor Residues and Chi Angles in PDB 7T6I and PDB 7ZFR. The image displays the models from PDB 7T6I and 7ZFR, showing both the experimental and modeled structures. The core anchor residues are highlighted, with positions 12, 9, 7, and 4 for 7T6I and positions 2, 5, 7, and 10 for 7ZFR. The chi angles of the sidechains for these anchor residues are shown, with the angles for 7T6I depicted in blue and those for 7ZFR in black. The chi angles are labeled adjacent to the respective bonds, emphasizing the conformational geometry of the key residues in both experimental and modeled structures.

Table S1. Torsion angles of 7T6I.

Residue	Phi	Psi	Chi1	Chi2	Chi3	Chi4
PRO 1	None	24.17	-9.67	-23.03	None	None
VAL 2	-122.00	137.15	162.54	None	None	None
ALA 3	-62.82	-53.24	None	None	None	None
ASP 4	-110.69	89.75	-93.91	-73.01	None	None
ALA 5	-94.14	142.56	None	None	None	None
VAL 6	-88.90	139.40	-58.18	None	None	None
ILE 7	-73.19	156.16	61.95	None	None	None
HIS 8	-112.94	136.82	-71.74	-64.12	None	None
ALA 9	-94.48	119.21	None	None	None	None
SER 10	-123.64	148.36	80.86	None	None	None
GLY 11	-102.07	165.85	None	None	None	None

LYS 12	-80.95	169.30	67.99	168.29	148.59	-76.38
GLN 13	-136.28	164.92	-47.49	-73.99	-14.33	None
MET 14	-61.77	144.65	-119.68	-179.17	72.35	None
TRP 15	-64.70	151.84	174.82	89.19	None	None
GLN 16	-63.99	None	80.86	-102.28	9.56	None

Table S2. Torsion angles of 7T6I modeled by PANDORA.

Residue	Phi	Psi	Chi1	Chi2	Chi3	Chi4
PRO 1	None	-17.71	7.65	-25.36	None	None
VAL 2	-93.079	126.13	178.60	None	None	None
ALA 3	-132.77	140.88	None	None	None	None
ASP 4	-104.76	134.32	-53.22	14.71	None	None
ALA 5	-74.47	153.65	None	None	None	None

VAL 6	-101.56	146.65	-58.28	None	None	None
ILE 7	-84.94	152.94	67.05	None	None	None
HIS 8	-107.20	135.16	173.92	-97.22	None	None
ALA 9	-94.76	122.82	None	None	None	None
SER 10	-99.50	148.59	-64.57	None	None	None
GLY 11	-107.22	146.71	None	None	None	None
LYS 12	-89.34	153.34	-74.52	179.28	-71.97	172.49
GLN 13	-132.45	148.25	-59.83	-60.81	120.83	None
MET 14	-149.17	-76.58	-71.037	-157.55	-79.025	None
TRP 15	-98.58	162.78	54.91	72.23	None	None
GLN 16	-106.53	None	-180.00	65.76	-100.10	None

Table S3. Torsion angles of 7ZFR.

Residue	Phi	Psi	Chi1	Chi2	Chi3	Chi4
ILE 1	None	-67.04	163.86	None	None	None
GLU 2	-124.70	132.59	-94.10	177.40	-51.90	None
PHE 3	-93.90	143.05	-63.45	-31.18	None	None
VAL 4	-78.45	158.54	-82.83	None	None	None
PHE 5	-75.25	146.77	-76.30	-27.24	None	None
LYS 6	-90.87	139.80	-59.14	-162.16	145.07	142.16
ASN 7	-112.20	111.71	-58.47	87.74	None	None
LYS 8	-112.76	-178.19	-55.40	-58.59	176.41	162.27
ALA 9	-150.72	158.95	None	None	None	None
LYS 10	-70.10	145.06	-50.31	128.95	54.02	166.37
GLU 11	-131.86	142.05	-64.13	-62.58	-12.53	None

**LEU 12**	**-78.49**	**None**	**-62.62**	**155.75**	**None**	**None**
**GLN 13**	**-132.45**	**148.25**	**-59.83**	**-60.81**	**120.87**	**None**
**MET 14**	**-149.17**	**-76.58**	**-71.03**	**-157.55**	**-79.02**	**None**
**TRP 15**	**-98.58**	**162.78**	**54.91**	**72.23**	**None**	**None**
**GLN 16**	**-106.52**	**None**	**-180.00**	**65.76**	**-100.10**	**None**

Table S4. Torsion angles of 7ZFR modeled by PANDORA.

**Residue**	**Phi**	**Psi**	**Chi1**	**Chi2**	**Chi3**	**Chi4**
**ILE 1**	**None**	**161.71**	**64.45**	**None**	**None**	**None**
**GLU 2**	**-121.96**	**136.20**	**-61.26**	**161.73**	**8.93**	**None**
**PHE 3**	**-76.64**	**152.20**	**-64.53**	**-85.24**	**None**	**None**
**VAL 4**	**-102.04**	**146.83**	**-57.56**	**None**	**None**	**None**
**PHE 5**	**-87.80**	**157.99**	**74.68**	**-58.96**	**None**	**None**

LYS 6	-115.10	141.72	-66.84	171.32	169.32	-179.94
ASN 7	-96.19	123.05	59.08	-23.28	None	None
LYS 8	-99.50	150.55	-63.69	-60.82	-180	-68.57
ALA 9	-108.854698181152	147.760589599609	None	None	None	None
LYS 10	-91.68	153.49	-73.79	175.38	-75.73	179.96
GLU 11	-133.73	131.44	-156.61	50.20	69.78	None
LEU 12	-80.83	None	-171.85	73.89	None	None
GLN 13	-132.45	148.25	-59.83	-60.81	120.87	None
MET 14	-149.17	-76.58	-71.03	-157.55	-79.025	None
TRP 15	-98.58	162.78	54.91	72.23	None	None
GLN 16	-106.52	None	-180.00	65.76	-100.10	None
